# Parallel Connected Generative Adversarial Network with Quadratic Operation for SAR Image Generation and Application for Classification

**DOI:** 10.3390/s19040871

**Published:** 2019-02-19

**Authors:** Chu He, Dehui Xiong, Qingyi Zhang, Mingsheng Liao

**Affiliations:** 1 Electronic and Information School, Wuhan University, Wuhan 430072, China; dhui.xiong@gmail.com (D.X.); zhqy@whu.edu.cn (Q.Z.); 2 Collaborative Innovation Center of Geospatial Technology, 129 Luoyu Road, Wuhan 430079, China; liao@whu.edu.cn; 3 State Key Laboratory for Information Engineering in Surveying, Mapping and Remote Sensing, Wuhan University, Wuhan 430079, China

**Keywords:** Synthetic Aperture Radar (SAR), image classification, Generative Adversarial Network (GAN), quadratic operation

## Abstract

Thanks to the availability of large-scale data, deep Convolutional Neural Networks (CNNs) have witnessed success in various applications of computer vision. However, the performance of CNNs on Synthetic Aperture Radar (SAR) image classification is unsatisfactory due to the lack of well-labeled SAR data, as well as the differences in imaging mechanisms between SAR images and optical images. Therefore, this paper addresses the problem of SAR image classification by employing the Generative Adversarial Network (GAN) to produce more labeled SAR data. We propose special GANs for generating SAR images to be used in the training process. First, we incorporate the quadratic operation into the GAN, extending the convolution to make the discriminator better represent the SAR data; second, the statistical characteristics of SAR images are integrated into the GAN to make its value function more reasonable; finally, two types of parallel connected GANs are designed, one of which we call PWGAN, combining the Deep Convolutional GAN (DCGAN) and Wasserstein GAN with Gradient Penalty (WGAN-GP) together in the structure, and the other, which we call CNN-PGAN, applying a pre-trained CNN as a discriminator to the parallel GAN. Both PWGAN and CNN-PGAN consist of a number of discriminators and generators according to the number of target categories. Experimental results on the TerraSAR-X single polarization dataset demonstrate the effectiveness of the proposed method.

## 1. Introduction

### 1.1. Background

Synthetic Aperture Radar (SAR) has gained immense popularity for its unique imaging capabilities. SAR provides high-resolution images independent of daylight, cloud coverage, and almost all weather conditions [1]. SAR images are useful for a multitude of applications, including remote sensing of the Earth’s surface, crop identification in agriculture, and flood mapping for disaster monitoring. As a result, SAR image classification has received extensive attention since 2000, when a reasonable number of SAR orbital systems became available.

In the field of image classification, feature extraction and feature selection are important steps. Various approaches have been proposed in the literature. Dudczyk et al. [2,3] studied SAR radar from the perspective of phase unwrapping and unintentional radiation, providing some ideas for the selection and extraction of SAR features. Zhao et al. [4] extracted the wavelet-invariant moment feature to indicate the SAR targets. Patnaik et al. [5] presented an SAR automatic target recognition system based on the minimum noise and correlation energy distortion-invariant filter. Park et al. [6] utilized the pixels and the projected length of the SAR target as the discriminative features. The magnitudes of the 2D DFT coefficients were used as fine features in [7]. The regional features and the predicted scattering center features were matched for SAR image classification in [8]. The pseudo-Zernike moments were adopted for target recognition in [9]. Dong et al. [10] explored an approach for SAR image classification via the sparse representation of monogenic signals. However, all of these methods rely heavily on hand-crafted features, which may suffer from poor adaptability.

In the early years, neural networks were widely applied to remote sensing imagery classification [11,12,13,14,15]. In recent years, methods [16,17] based on deep learning have received widespread attention and achieved appealing results. CNNs are one of the most typical network structures in the deep learning field. They have achieved great success in the field of target recognition. Compared with traditional target recognition algorithms, CNNs have the advantage of avoiding the complicated pre-processing of images. Moreover, in contrast with methods based on hand-crafted feature extraction, CNN-based methods can automatically learn features from large amounts of data. for the standard ImageNet dataset, many successful models are based on CNNs. AlexNet, proposed by Krizhevsky et al. [18], has attracted substantial attention by achieving superior image classification accuracy on the ImageNet dataset. Since then, VGG-16-Net [19], GoogLeNet [20], and ResNet [21], with more complex CNN architectures, have gradually improved the recognition rate. Driven by massive amounts of data, deep CNNs have won great popularity. Unfortunately, due to the different imaging mechanisms of SAR, it is difficult to label images. Insufficient labeled SAR data become an obstacle to the use of CNNs in SAR target recognition.

### 1.2. Problems and Motivation

For the limited labeled SAR data problem, most deep CNN-based approaches [22,23] have attempted to improve the network structure instead of obtaining more training data. Generative Adversarial Nets (GANs) [24] have an excellent performance in data generation and can provide additional data to augment the utilized dataset. Therefore, applying GANs to generate more data is a potential approach to addressing the overfitting problem when the training data are inadequate. Zheng et al. [25] used GANs to generate unlabeled samples to improve the person re-identification baseline in vitro. Qian et al. [26] designed a Pose-Normalization GAN (PN-GAN) to address the lack of cross-view paired training data problem. Merkle et al. [27] investigated the use of GANs to predict optical patches from SAR patches and vice versa. Hughes LH et al. [28] generated artificial hard negative SAR patch examples to improve the training of an SAR-optical matching CNN. Dimitrios Marmanis et al. [29] tried to use artificial generation of big data to improve image classification, but the experiments have not yet led to conclusive results. Although these methods provide a feasible way to solve the problem of limited labeled SAR image classification, the current GANs are still not effective at solving this problem.

The original GAN is sometimes very unstable and faces difficulties in converging during the training process. Deep Convolutional GANs (DCGANs) [30] optimize the network structure and improve the stability of training, but the samples generated by DCGAN usually lack diversity because of the vanishing gradient problem when using the gradient descent method to train DCGAN. To address the vanishing gradient problem, the Wasserstein GAN (WGAN) [31] replaced the Jensen–Shannon (JS) divergence with the Earth-Mover (EM) distance [32] for evaluating the distribution between the real data and the generated data. WGAN made significant progress toward the stable training of GANs, but it could still generate low-quality samples or fail to converge in certain settings. WGANs with Gradient Penalty (WGAN-GP) [33] improved upon WGAN, thereby providing higher quality samples, more stable training, and faster convergence. Nevertheless, the generated samples still cannot satisfy the requirements of SAR image classification.

In practice, the samples generated by DCGAN present fine-structure features, while the samples generated by WGAN-GP are rich in diversity. Therefore, it may be a good choice to utilize both advantages of DCGAN and WGAN-GP. In a classification task, the target contains multiple categories. In supervised learning, data need to be labeled during the training process. Unfortunately, data generated by ordinary GANs are often not automatically labeled. This may bring new problems to the expansion of the labeled training set. Hence, GAN needs to be redesigned on the overall structure. In addition, ordinary GANs do not consider the characteristics of SAR images in the generation process. Therefore, the value function of GAN can be combined with some SAR mechanisms to make the generated SAR image more realistic. Furthermore, considering that the convolution in the discriminator is only a first-order operation without a strong description of the characteristics of SAR image multiplicative speckles, an expansion of the convolution with the quadratic form can enhance the discriminator’s performance. In this way, a special GAN can be developed for SAR image generation.

### 1.3. Contributions and Structure

In this paper, special GANs are designed to generate additional labeled SAR images for training and thus improve the classification accuracy. The main contributions are summarized as follows:

First, considering the unique imaging mechanism of SAR such as the characteristics of multiplicative speckles in SAR images, we design a GAN more suitable for generating SAR images, which is more helpful to the SAR classification. On the one hand, the quadratic operation is extended to the convolution in the GAN to improve the representation capability of the discriminator. On the other hand, merging the statistical properties of SAR images into the value function of GAN makes the generated SAR images more realistic and suitable for classification.

Second, on the overall structure, two types of GANs (WPGAN and CNN-PGAN) are proposed for SAR image generation. A significant part of our model consists of a series of discriminators and generators connected in parallel according to the number of SAR image categories. Thus, it is not necessary to manually label the generated data. On the other hand, a manual visual selection is necessary to validate the generated data. In PWGAN, for a single discriminator and generator, the network structure is in line with DCGAN, and the discrimination function is similar to that in WGAN-GP; in CNN-PGAN, a shared discriminator is introduced to the network. We validate that the samples produced by the proposed method can help to improve the accuracy of SAR image classification.

Third, we also demonstrate that the number of generated SAR images used to augment the training SAR dataset has a strong influence on the classification result. Instead of using all the generated SAR images, a certain number of generated samples are carefully selected.

The remainder of this paper is organized as follows. In Section 2, we give an introduction to the related work on SAR image classification and GANs. In Section 3, the details of the methodology are described. In Section 4, results are presented, and Section 5 provides more discussions. Section 6 concludes this paper.

## 2. Related Work

In this section, we give a brief overview of previous studies related to SAR image classification with CNNs, and then, we discuss the relevant work on GANs.

### 2.1. SAR Image Classification with CNNs

**Figure 1:**
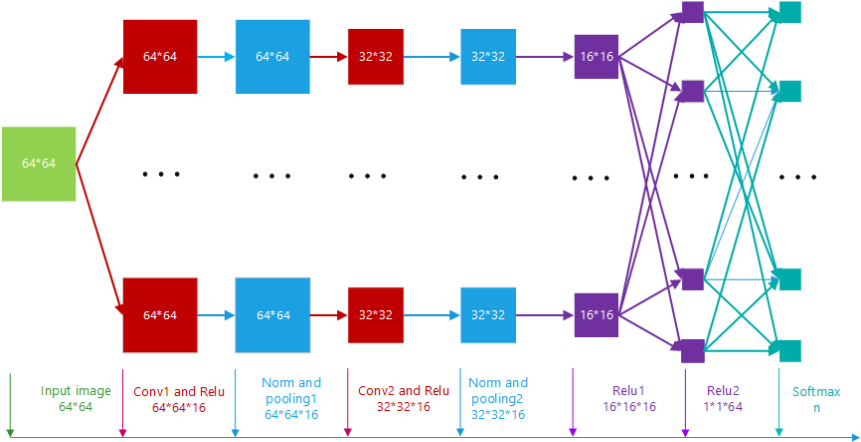
Assembled CNN architecture.

A basic CNN usually includes an input layer, several convolutional layers, several pooling layers, some fully-connected layers, and an output layer. The architecture of the CNN adopted in our paper is shown in Figure 1. Various methods based on CNNs have been proposed for SAR image classification. Chen et al. [34] first introduced a single convolutional layer to extract SAR image feature representation effectively with unsupervised learning using randomly-sampled SAR target patches in 10-class classification tasks. Danilla et al. [35] designed a network architecture consisting of a sequence of convolutional layers with fully-connected layers at the end, where the convolutional layers perform speckle filtering and feature extraction. Morgan et al. [36] utilized a structure of three convolutional layers and one fully-connected layer, thereby improving the accuracy of the SAR image classification. Wilmanski et al. [37] proposed a different learning algorithm to train CNNs; they discovered that, compared with Stochastic Gradient Descent (SGD) and AdaGrad [38], AdaDelta [39] could achieve substantially higher learning rates of the hyper-parameters. Chen et al. [40] proposed a novel CNN, which only consists of sparsely-connected layers, without fully-connected layers being used, to reduce the independent trainable parameters. Huang et al. [41] introduced a transfer learning-based approach, therein exploring the appropriate source data from which to transfer. Instead of using the existing model trained with the labeled ImageNet dataset in most literature, the unlabeled SAR scene imagery is utilized to train the convolutional layers to be transferred to SAR recognition tasks later. GAN can be used to generate a large amount of data, which provides a feasible way to solve the problem of insufficient labeled SAR images for classification. However, as described in Section 1.2, GAN has not achieved satisfactory results in improving the classification effect of SAR images. Compared with the previous work [29], we adjusted the internal structure of GAN to make the generated SAR image more SAR-like. At the same time, the proposed method can improve the classification accuracy of SAR images. Therefore, we try to use GAN to generate labeled SAR images to solve the problem of insufficient training data, instead of using other extra data or adopting a new network for classification.

### 2.2. Generative Adversarial Networks

#### 2.2.1. GANs

**Figure 2:**
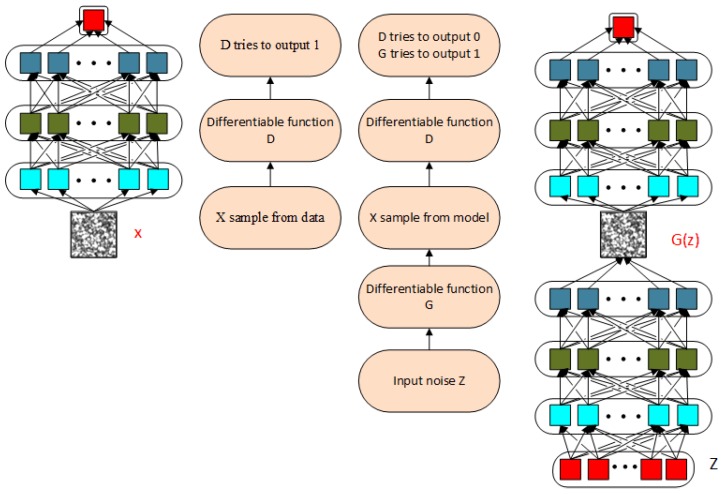
Schematic of a GAN.

The basic idea of GANs is inspired by the Nash equilibrium [24] in game theory. It assumes two game players: a generator and a discriminator. The generator attempts to generate new samples, which are expected to be consistent with the distribution of the real data, while the discriminator attempts to determine whether the input data are real or generated. Through competition with each other, the generation ability and the discrimination ability are improved. Figure 2 shows a schematic of the GAN. The functions *G* and *D* represent the generator and the discriminator, respectively. The inputs are random variable *z* and real data *x*. G(z) represents the generated samples. The competition between *G* and *D* can be described by the value function:

(1) $$\begin{array}{c} \begin{array}{c} \min_{G}\max_{D}V\left(G,D\right)=E_{x∼p_{data}\left(x\right)}\left[\log D\left(x\right)\right]+E_{z∼p_{z}\left(z\right)}\left[\log\left(1-D\left(G\left(z\right)\right)\right)\right], \end{array} \end{array}$$

where $p_{data}$ represents the distribution of real data and $p_{z}\left(z\right)$ is a simple fixed distribution of the latent variable *z*. GANs provide an attractive alternative to maximum likelihood techniques.

#### 2.2.2. DCGAN

The training process of the original GANs is often unstable and difficult to converge, resulting in a generator that produces nonsensical outputs. Radford et al. [30] first introduced DCGAN to generate images. Some optimizations were made in the network structure. The discriminator and the generator use strided and fractional-strided convolutions instead of pooling layers. In the discriminator, LeakyReLU activation is applied for all layers; in the generator, ReLU activation is used for all layers except the output. Batchnorm is adopted in both the discriminator and the generator. All fully-connected hidden layers are removed for deeper architectures. In general, DCGAN can produce very visually-appealing samples. However, the generated samples are similar in certain cases.

#### 2.2.3. WGAN-GP

The work in [24] noted that minimizing the value function also minimizes the JS divergence between $p_{data}$ and $p_{z}\left(z\right)$, which often leads to vanishing gradients. Wasserstein WGAN attempts to use the EM distance to replace the JS divergence. The value function of WGAN can be constructed using the Kantorovich–Rubinstein duality [42] to obtain:

(2) $$\begin{array}{c} \begin{array}{c} E_{z∼p_{z}\left(z\right)}\left[D\left(G\left(z\right)\right)\right]-E_{x∼p_{data}\left(x\right)}\left[D\left(x\right)\right]. \end{array} \end{array}$$

where *D* is the set of one-Lipschitz functions. In that case, under an optimal discriminator, minimizing the value function with respect to the generator parameters minimizes the EM distance:

(3) $$\begin{array}{c} \begin{array}{c} W\left(p_{data},p_{G(z)}\right)=\underset{\gamma \in \Pi \left(p_{data},p_{G(z)}\right)}{\inf}E_{(x,y)∼\gamma }\left[∥x-y∥\right], \end{array} \end{array}$$

where $\gamma \in \Pi (p_{data},p_{G(z)})$ denotes the set of all joint distributions γ(x,y) whose marginals are $p_{data}$ and $p_{G(z)}$, respectively. The developers of WGAN-GP observed that the weight clipping used in WGAN can lead to undesirable results and modified the value function by adding a gradient penalty item to obtain:

(4) $$\begin{array}{c} \begin{array}{c} E_{z∼p_{z}\left(z\right)}\left[D\left(G\left(z\right)\right)\right]-E_{x∼p_{data}\left(x\right)}\left[D\left(x\right)\right]+\lambda E_{z∼p(z)}\left[\left(\right\|\nabla_{z}D\left(G\left(z\right)\right){\left|{|}_{2}-1\right)}^{2}\right], \end{array} \end{array}$$

where λ is the penalty coefficient and $\nabla_{z}$ denotes the gradient function.

## 3. Materials and Methods

### 3.1. Problems’ Overview

SAR images generated by GAN can be used to expand the training set for improved classification. The ordinary GAN does not account for the characteristics of the SAR image when used to generate SAR images. Therefore, how to design a GAN that incorporates the characteristics of SAR images to generate SAR images suitable for classification is a problem that needs to be solved. In addition, an SAR image generated by the ordinary GAN may contain multiple categories, which is difficult to classify. At the same time, the generated SAR images are not labeled, so they cannot be directly used to expand the training set. Hence, how to make generated SAR images have clearer categories and be automatically labeled is the second problem we need to solve.

### 3.2. The Proposed GANs

To solve the first problem mentioned in Section 3.1, we extended the convolution of GAN by introducing a quadratic operation and made it become part of the G and D functions. In addition, we also incorporated the SAR statistics into the value function of GAN. To solve the second problem, parallel-connected GANs on the overall structure were designed. We will describe the proposed approach in detail in this section.

#### 3.2.1. Quadratic Operation

An SAR image is essentially a statistical signal. It is important to find a suitable statistical model to describe it. Table 1 shows the expressions obtained by the moment parameter estimation method for several common empirical distribution models and a priori distribution models.

In the table, $m_{1}$ and $m_{2}$ are first-order moments and second-order moments, respectively. Γ(.) is the gamma function. The expressions of $m_{1}$, $m_{2}$, Γ(.) are as shown in Equations (5)–(7).

(5) $$\begin{array}{c} \begin{array}{c} m_{1}=\sum_{i}p_{i}, \end{array} \end{array}$$

(6) $$\begin{array}{c} \begin{array}{c} m_{2}=\sum_{i}p_{i}^{2}, \end{array} \end{array}$$

(7) $$\begin{array}{c} \begin{array}{c} \Gamma \left(p\right)=\int_{0}^{+\infty }t^{p-1}e^{-t}dt. \end{array} \end{array}$$

It can be seen that in these statistical models, the parameter estimation formula is generally a diverse combination of $m_{1}$, $m_{2}$, and $m_{1}^{2}$. Therefore, considering the characteristics of multiplicative speckles in SAR images, it is a feasible solution to characterize SAR images by using quadratic terms. However, because the convolution in the ordinary GAN is only a first-order operation, the characteristics of the SAR image cannot be well described. Therefore, we extent the convolution by introducing the quadratic operation to improve signal representation. More formally, the convolution process can be expressed as:

(8) $$\begin{array}{c} \begin{array}{c} f_{1}\left(p\right)=\mathrm{wp}=w_{1}p_{1}+w_{2}p_{2}+\cdots +w_{n}p_{n}, \end{array} \end{array}$$

where **w** is the kernel of the convolution and **p** is the input signal. The quadratic term can be expressed as:

(9) $$\begin{array}{c} \begin{array}{c} f_{2}\left(p\right)=p^{T}\mathrm{Ap}=\left[p_{1},p_{2},\ldots ,p_{n}\right]\left[\begin{array}{cccc} a_{11} & a_{12} & \ldots & a_{1n}\\ a_{21} & a_{22} & \ldots & a_{2n}\\ \vdots & \vdots & \vdots & \vdots \\ a_{n1} & a_{n2} & \ldots & a_{nn} \end{array}\right]\left[\begin{array}{c} p_{1}\\ p_{2}\\ \vdots \\ p_{n} \end{array}\right]=\sum_{i=1}^{n}\sum_{j=1}^{n}a_{ij}p_{i}p_{j}, \end{array} \end{array}$$

where **A** is the corresponding coefficient matrix. We combine Equation (8) with Equation (9) to obtain an extended convolution, which is expressed as Equation (10):

(10) $$\begin{array}{c} \begin{array}{c} f\left(p\right)=f_{1}\left(p\right)+f_{2}\left(p\right)=\sum_{i=1}^{n}\sum_{j=1}^{n}a_{ij}p_{i}p_{j}+\sum_{i=1}^{n}w_{i}p_{i}. \end{array} \end{array}$$

**Figure 3:**
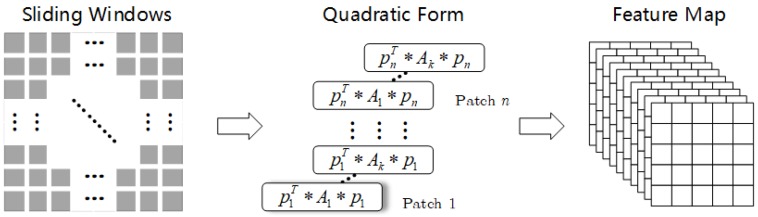
Quadratic operation.

The calculation of the quadratic form is similar to the convolution. The sliding window operation is used, as shown in Figure 3. Through the above operation, the GAN incorporates the quadratic term.

#### 3.2.2. Parallel Connected GANs

**Figure 4:**
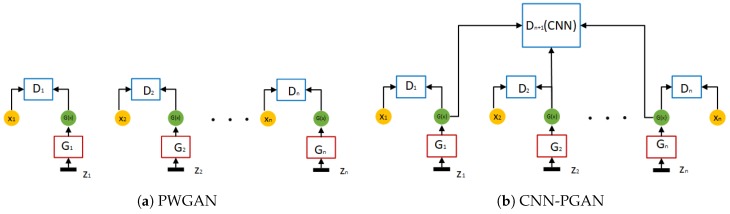
Two different architectures of our proposed GAN.

To illustrate the proposed GAN, we present a brief diagram in Figure 4. We designed two different types of GANs (PWGAN and CNN-PGAN), which are similar to each other in architecture, to generate a labeled SAR image. The structure of PWGAN is shown in Figure 4a, while CNN-PGAN is illustrated in Figure 4b. Usually, a normal GAN contains one generator and one discriminator, which can be used to generate different images. However, the images generated by this type of GAN often lack a clear categorization. Hence, in our two types of GANs, several generators and discriminators connected in parallel were adopted according to the number of categories in the targets. In the PWGAN, if the targets have ncategories, then n generators and n discriminators are applied. Each category has a separate generator and a separate discriminator. Therefore, SAR data can be generated with a kind of label. Therefore, there is no need for manual labeling. In the structure of CNN-PGAN, a shared discriminator is added to the network. This type of structure is related to that adopted in [43]. Let {$G_{1}$, $G_{2}$, …, $G_{n}$} denote the group of generators and {$D_{1}$, $D_{2}$, …, $D_{n}$} denote the group of discriminators. The function of the generator and the discriminator ({$G_{1}$, $D_{1}$}, {$G_{2},D_{2}$}, …, {$G_{n}$, $D_{n}$}) is to make the generated images have individual image features.

#### 3.2.3. PWGAN

This type of GAN employs an architecture similar to DCGAN and adopts a value function derived from WGAN-GP. In other words, DCGAN and WGAN-GP are combined to generate SAR images, which can maintain structural consistency with the real images and provide rich sample diversity. In addition to adopting the special architecture described above, we modified the value function by integrating SAR image characteristics into the GAN. Specifically, a new term that contains statistical information is added to the value function. In this way, the value function for each category consists of two different parts: a WGAN-GP loss part and a statistical average loss part. More formally, the total value function of the first type of proposed GAN can be expressed as:

(11) $$\begin{array}{c} \begin{array}{c} \min_{\left\{G_{1},G_{2}\ldots G_{n}\right\}}\max_{\left\{D_{1},D_{2}\ldots D_{n}\right\}}V\left(G,D\right)=\min_{G_{1}}\max_{D_{1}}V\left(G,D\right)+\min_{G_{2}}\max_{D_{2}}V\left(G,D\right)+\ldots +\min_{G_{n}}\max_{D_{n}}V\left(G,D\right). \end{array} \end{array}$$

where the items on the right side in Equation (11) uniformly correspond to:

(12) $$\begin{array}{c} \begin{array}{c} \min_{G_{k}}\max_{D_{k}}V\left(G,D\right)=\mu L_{WGAN-GP}+\delta L_{statistical-aver}. \end{array} \end{array}$$

where μ,δ are hyperparameters, k∈{1,2,…,n}. In particular,

(13) $$\begin{array}{c} \begin{array}{c} L_{WGAN-GP}=E_{z∼p_{z}\left(z\right)}\left[D\left(G\left(z\right)\right)\right]-E_{x∼p_{data}\left(x\right)}\left[D\left(x\right)\right]+\lambda E_{z∼p(z)}\left[\left(\right\|\nabla_{z}D\left(G\left(z\right)\right){\left|{|}_{2}-1\right)}^{2}\right]. \end{array} \end{array}$$

The right side of the equation is the same as in Equation (4).

(14) $$\begin{array}{c} \begin{array}{c} L_{statistical-aver}=\left|E\left[I_{G}\right]-E\left[I_{R}\right]\right|, \end{array} \end{array}$$

Equation (14) is the absolute value of the statistical average difference between the generated images and the real data, where $I_{G}$ and $I_{R}$ represent the generated image and the real image, respectively.

#### 3.2.4. CNN-PGAN

CNN-PGAN utilizes the same structure as DCGAN. However, compared to the first approach, the second approach does not combine WGAN-GP with DCGAN, but a shared discriminator $D_{n+1}$ is designed to determine the category attribute. This modification is more helpful for SAR image classification. In this paper, $D_{n+1}$ is a pre-trained CNN whose parameters are not updated during the training. It can be used to calculate the classification loss, which is a part of the GAN value function. The pre-trained CNN possesses the same structure as the CNN in Figure 1 trained with the original training set. A new term that contains statistical information is also added to the value function, similar to the first type. Hence, the value function consists of three different parts: a DCGAN loss part, a shared discriminator loss part, and a statistical average loss part. Equation (11) for the first type can be changed to:

(15) $$\begin{array}{c} \begin{array}{c} \begin{array}{c} \min_{\left\{G_{1},G_{2}\ldots G_{n}\right\}}\max_{\left\{D_{1},D_{2}\ldots D_{n},D_{n+1}\right\}}V\left(G,D\right)=\min_{G_{1}}\max_{\left\{D_{1},D_{n+1}\right\}}V\left(G,D\right)+\\ \min_{G_{2}}\max_{\left\{D_{2},D_{n+1}\right\}}V\left(G,D\right)+\ldots +\min_{G_{n}}\max_{\left\{D_{n},D_{n+1}\right\}}V\left(G,D\right), \end{array} \end{array} \end{array}$$

where the items on the right side in Equation (15) can be expressed as:

(16) $$\begin{array}{c} \begin{array}{c} \min_{G_{k}}\max_{\left\{D_{k},D_{n+1}\right\}}V\left(G,D\right)=\mu L_{DCGAN}+\delta L_{statistical-aver}+\eta L_{D_{n+1}}. \end{array} \end{array}$$

in which η is a hyperparameter and $L_{D_{n+1}}$ is the shared discriminator loss, which comes from the pre-trained CNN. Because DCGAN takes the same value function as the original GAN, $L_{DCGAN}$ is calculated by Equation (1).

### 3.3. Materials

**Figure 5:**
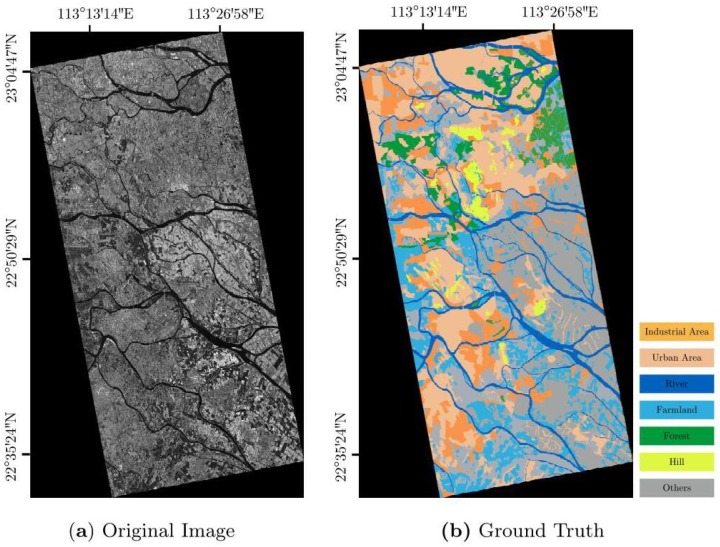
DataSet1 (7 categories): single-polarized (VV) data acquired by TerraSAR-X over Guangdong, China (intensity image).

**Figure 6:**
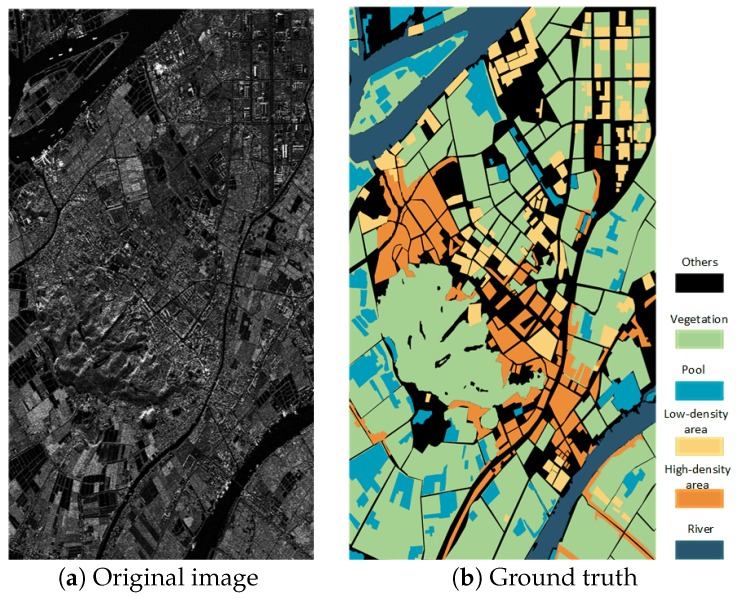
DataSet2 (there are 6 categories, but only 5 are considered).

To evaluate the proposed methods, we used a dataset from a full scene acquired by TerraSAR-X with single polarization (VV channel) over Guangdong Province, China, on 24 May 2008. The processing level of this scene was 2. Multi-looking processing was done by the data provider. Geocoding was done by SNAP software provided by ESA. The conversion to intensity backscattering was also done by SNAP software. We used optical images on Google Maps for the same period as a reference for manual annotation data. The data consisted of 7 categories, i.e., Industrial area, Urban area, River, Farmland, Forest, Hill, and others, as shown in Figure 5. We used a 64 × 64 window to slide across the image with a stride of (64, 64), cutting the entire scene into many 64 × 64 pixel subgraphs. There was no overlap between the images and no superposition for each categories. For each category, we randomly selected 160 different images, of which 128 were used for training and the others were used as the test set. The training images and test images were also randomly selected. The dataset was called DataSet1. To further verify our approach, we selected one large part of the full scene as the new dataset shown in Figure 6. This new dataset contained 6 categories, i.e., others, river, pool, vegetation, low-density area, and high-density area. We also cut the scene into many 64 × 64 pixel subgraphs. Except for the others category, we randomly selected 160 different images for each category. A total of 128 images were used for training the dataset, and the other 32 images were used for testing. We called this new dataset DataSet2.

### 3.4. Framework and Setting

**Figure 7:**
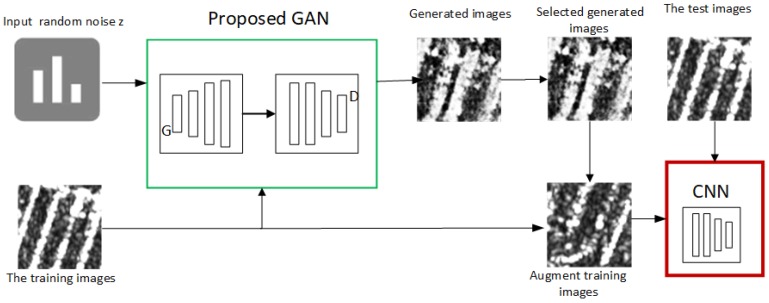
Overview of our framework.

Figure 7 shows the framework we used for SAR image classification. The network mainly consisted of two parts: a GAN and a CNN. The GAN is used to generate new SAR images to expand the training dataset, while the function of the CNN is to classify the SAR images. The system workflow can be briefly summarized as follows. First, the training images and random noise were used as the input of our proposed GANs. Second, a certain number of high-quality generated images were manually selected to augment the training data. The steps to select the generated data manually were as follows: (1) We usually selected the images with good visual effects (similar to the original images), which were generated by one or several entire epochs; (2) If the selected generated data contained a small number of “bad” images (not similar to the original images), these images would be replaced by “good” images (similar to the original images) generated by adjacent epochs. Finally, we trained the CNN using the augmented training data and verified the CNN on the test dataset.

As shown in Figure 7, the framework used for SAR target classification was composed of a GAN and a CNN. The architecture of the CNN is illustrated in Figure 1. The CNN contained 8 layers, i.e., one input layer, one output layer, two convolutional layers, two max pooling layers, and two fully-connected layers. The learning rate was set to 0.0001. Table 2 summarizes the settings of the CNN.

The architecture of the proposed GAN is shown in Figure 4. For DataSet1, there were 7 different categories of SAR images. Therefore, for the first type of proposed GAN, 7 generators and 7 discriminators were adopted; for the second type of proposed GAN, one additional discriminator was employed, which was a pre-trained CNN with a test accuracy of 60%. The settings of ${\left\{G_{k},D_{k}\right\}}_{k\in \{1,\ldots ,7\}}$ mainly followed [30]. Moreover, we set the penalty coefficient λ to 10 in Equation (13) and the other hyperparameters μ,δ and η to 1 in Equation (16). Adam [44] was replaced by RMSProp. For DataSet2, there were 5 different categories of SAR images. Compared to DataSet1, the settings of correlational parameters differed only in the number of generators and discriminators.

## 4. Experimental Results

Experiments were performed on four NVIDIA Titan X’s using Tensorflow. It took four hours to train the proposed GANs in the experiment. In this subsection, the experimental results will be presented from different perspectives. In order to avoid the large randomness of one experiment, we conducted 10 executions of each network architecture to obtain the average results.

### 4.1. Generated Images

Table 3 shows the original images of DataSet1 and the corresponding generated images by the proposed PWGAN; Table 4 demonstrates the original images of DataSet2 and the corresponding generated images by the proposed CNN-PGAN. Classes 0–6 represent the seven categories, i.e., Forest, Hill, Industrial area, Farmland, others, River, and Urban area for DataSet1; while Classes 0–4 represent the five categories, i.e., Vegetation, Pool, River, Low-density area, and High-density area for DataSet2. For a better presentation, three images were selected for each category. The results show that PWGAN and CNN-PGAN can generate images visually similar to the original images.

### 4.2. Results on Different Numbers of Generated Images for Training CNN

GAN can generate thousands of images. Not all generated images are suitable for augmenting the training set. Therefore, we should choose an appropriate number of high-quality generated images for training. However, it is difficult to find an objective standard to evaluate the quality of the generated images. In the experiment, the images with better visual effects were selected after the training of GAN had converged. Generally, an epoch produced 64 images. For the comparison test, we selected 32, 64, 128, and 256 images, which were produced by half an epoch, an epoch, two epochs, and four epochs. As for which epochs we chose, this needed to be based on the visual effects of the images they produced.

**Figure 8:**
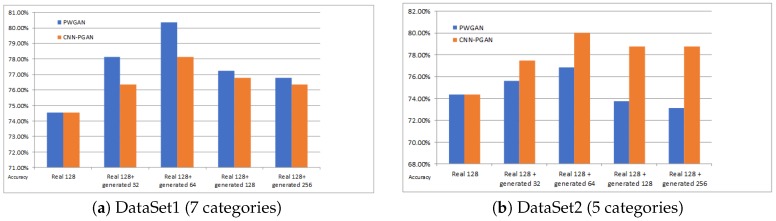
The accuracy for different amounts of generated data for training on DataSet1 and DataSet2. The blue bar and the yellow bar indicate the classification accuracies of PGAN and CNN-PGAN, respectively. The first column is the classification result on the real 128 training images. The second, third, fourth, and fifth columns are the results of training data augmentation using 32, 64, 128, and 256 generated images, respectively.

The experimental results on DataSet1, as shown in Figure 8a, indicate that the classification accuracy of the proposed methods was improved. Compared with the original training dataset, the accuracy of PWGAN was increased by 3.58% (from 74.55% to 78.13%), 5.81% (from 74.55% to 80.36%), 2.68% (from74.55% to 77.23%), and 2.24% (from 74.55% to 76.79%), respectively. The accuracy of CNN-PGAN was increased by 1.79% (from 74.55% to 76.34%), 3.58% (from 74.55% to 78.13%), 2.24% (from74.55% to 76.79%), and 1.79% (from 74.55% to 76.34%), respectively. Figure 8b presents the classification accuracy for different numbers of generated images used for training on DataSet2. Compared with the original training dataset, the accuracy of CNN-PGAN was increased by 3.12% (from 74.38% to 77.50%), 5.62% (from 74.38% to 80.0%), 4.25% (from 74.38% to 78.63%), and 3.65% (from 74.38% to 78.03%), respectively. Meanwhile, the accuracy of PWGAN was increased by 1.25% (from 74.38% to 75.63%) and 2.50% (from 74.38% to 76.88%) when the original training data were augmented by 32 and 64 generated images. However, when 128 and 256 generated images were used, the result was worse than with the original data. Obviously, the number of generated images and the classification results did not show a positive correlation. Therefore, only using suitable number of generated images to augment the training dataset can result in the best classification result. It seems that the images generated in one epoch were more helpful for improving the classification accuracy than in other epochs.

### 4.3. Results on Different Numbers of Real Images for Training the Entire Network

**Figure 9:**
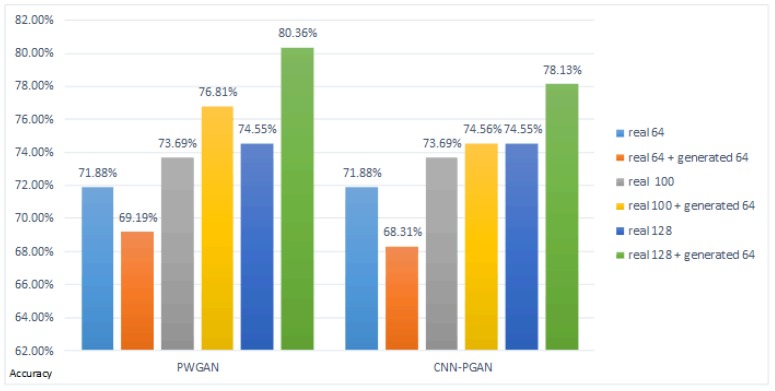
The classification result of different numbers of real images for training the entire network on DataSet1.

To determine what would happen if more (or less) real images were available, another experiment was carried out. In the experiment, there were 64, 100, and 128 real images randomly selected as benchmarks for training the entire network, respectively. In each case, 64 generated images were selected to expand the CNN training set as a comparison. The result is shown in Figure 9. It can be seen that the classification accuracy was not always improved. The accuracy of PWGAN was decreased by 2.69% (from 71.88% to 69.19%) and the accuracy of CNN-PGAN was decreased by 3.57%(from 71.88% to 68.31%) when only 64 real images were available. Therefore, when the number of real images for training GAN was too small, it was difficult for GAN to generate high quality samples to augment the training set of CNN.

### 4.4. Comparison with the Simple Data Augmentation Technique

**Figure 10:**
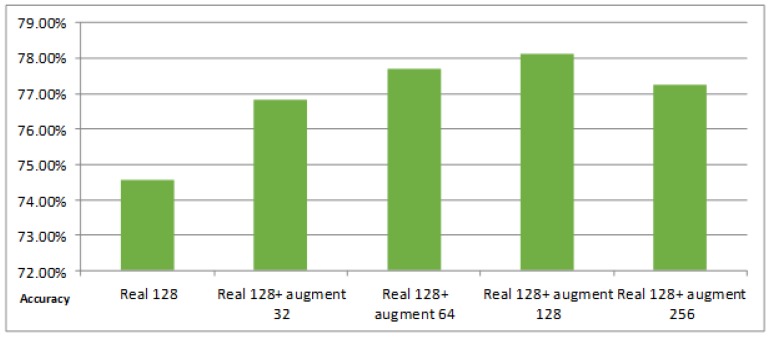
The classification result of different numbers of augmented training data for DataSet1. The first column is the classification result on the real 128 training images. The second, third, fourth, and fifth columns are the results of training data augmentation using 32, 64, 128, and 256 images augmented by the simple augmentation strategy, respectively.

**Figure 11:**
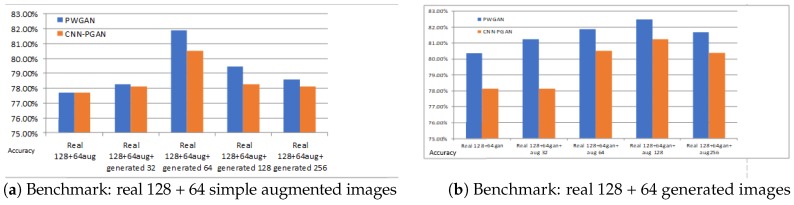
The accuracy for different training datasets. The blue bar and the yellow bar indicate the classification accuracies of PWGAN and CNN-PGAN, respectively. (**a**) The first column is the classification result on the training dataset (128 real images and 64 augmented images). The second, third, fourth, and fifth columns are the results of training data augmentation using 32, 64, 128, and 256 generated images, respectively; (**b**) The first column is the classification result on the training dataset (128 real images and 64 augmented images). The second, third, fourth, and fifth columns are the results of training data augmentation using 32, 64, 128, and 256 augmented images, respectively. The test set is always the same.

There are many simple augmentation techniques to augment the training set, such as the introduction of random Gaussian noise, rotation, mirroring, flipping, etc. To compare with these methods, we used the Augmentor toolkit in Python to augment the training data. Thirty two, 64, 128, and 256 images augmented by the Augmentor toolkit were used for the experiments. The experimental result is presented in Figure 10, which indicates that the classification accuracies were improved by adding different numbers of augmented data. Hence, the simple data augmentation strategy was effective. To further verify the effectiveness of the proposed methods, we set up two groups of comparison experiments. In the first group of experiments, 128 original images and 64 images augmented by the Augmentor toolkit on the training set were used as benchmarks, and 32, 64, 128, and 256 generated images were added for comparison, while in the second group, 128 original images and 64 generated images were used as benchmarks and 32, 64, 128, and 256 images augmented by Augmentor toolkit were added for contrast. The results are shown in Figure 11. It can been seen that the proposed methods can further improve the classification accuracy with the simple data augmentation strategy. In other words, our approach can be used with other augmentation strategies to augment the training dataset to improve the classification efficiency. However, data augmentation requires extra time.

### 4.5. Comparison of the Classification Results Using Different Methods

Furthermore, we validated the proposed methods by comparing them with CNN without the proposed GAN part, AlexNet, DCGAN, and WGAN. In addition, we tried to compare the proposed methods with GoogLeNet and ResNet; however, GoogLeNet and ResNet are too deep, and there are too many parameters to train. When they were used on our limited training dataset, the networks could not converge. Moreover, transfer learning had been taken into consideration. On the one hand, the CNN and GAN networks adopted already used fine-tuning. On the other hand, transfer learning on the GoogLeNet and ResNet failed. In the comparison experiment, the setting of AlexNet mainly followed [18]. The results are given in Table 5 and Table 6. The classification accuracy of CNN and AlexNet was not much different. For DataSet1, the classification accuracy of the DCGAN was 73.21%, which was lower than that of the CNN. WGAN-GP could achieve an increase of 2.68% (from 74.55% to 77.23%). Compared with the above two methods, our methods obtained better classification results. The proposed PWGAN achieved the highest classification accuracy, and the proposed CNN-PGAN also improved the accuracy from 74.55% to 78.13%. The classification result on DataSet2 was basically consistent with the results on DataSet1. The two proposed types of GANs could achieve 2.40% and 5.62% improvements compared with CNN. Table 7, Table 8 and Table 9 show the classification confusion matrix of CNN, PWGAN, and CNN-PGAN on DataSet1, respectively. The results clearly indicate that our two proposed types of GANs could generate high-quality SAR images for augmenting training datasets.

## 5. Discussion

This paper has presented an effective classification method based on GANs for limited quantities of labeled SAR images. In the experiment on a TerraSAR dataset, the following interesting results were revealed.

### 5.1. The Effects of GANs

GANs can generate thousands of samples that can be used to expand the training data, but not all the GANs are suitable for generating SAR images. Table 5 and Table 6 illustrate that the proposed GANs were very helpful for the classification of SAR images. Figure 11 demonstrates that the proposed methods were also effective on the simple data augmentation strategy. In other words, our approach can be used with other augmentation strategies to augment the training dataset to improve the classification efficiency. Obviously, the proposed GANs were also compatible with other methods and different data. The experimental results in Figure 9 indicate that when there were too little real data available, it was difficult for GAN to generate high-quality samples, which cannot help improve the classification accuracy of CNN. This reflects that the proposed GANs can exert their effects under certain conditions. The proposed methods can be further improved in subsequent research.

### 5.2. The Number of Generated Images for Training

Figure 8 indicates that the number of images generated for training strongly influenced the classification results. It also shows that not all data generated by the proposed GAN were helpful for classification, nor was it the case that the more data were generated, the better the classification result was. From another perspective, it reflects that the diversity of generated images was limited. In the comparison experiment, the best classification result was obtained by generating 64 images, but there may be other better choices. A possible reason why using 64 artificially-generated images led to the best results in the comparison experiment may be that the proposed GANs generated 64 images per epoch, which may own the best diversity. However, the 64 images selected may be not generated by one epoch for all the experiments. Thus, we cannot draw a definitive conclusion. Therefore, we did not find the ideal number of artificially-generated images. Therefore, choosing the most suitable quantity is a challenging problem that needs to be solved in our follow-up study.

### 5.3. The Criteria for Evaluating the Quality of Generated Images

Generally, there is no objective criterion for evaluating the quality of the generated images. This brings great difficulties to the selection of generated images. If the selected sample is not good enough, it will cause serious interference in the classification. In the experiment, we manually selected the generated images with good visual effects as shown in Table 3 and Table 4, which may strongly affect the stability of the experimental results. Since we did not need to select a large number of generated data to augment the training dataset in the experiment, it did not consume much time. However, the manual selection of data was indeed a major drawback in the proposed system. Establishing a reasonable and objective evaluation standard is an urgent problem to be solved.

## 6. Conclusions

In this paper, an effective classification scheme based on GANs for limited quantities of labeled SAR images has been proposed. The main contribution is that we attempted to use GANs to solve the SAR image classification problem. We extended the convolution of the GAN with a quadratic term and incorporated SAR image features into the value function. In addition, we designed two types of GANs to generate SAR images that were more suitable for classification tasks. The experiments conducted on the TerraSAR-X dataset showed that by adding a certain number of SAR images generated by the proposed GAN to expand the training dataset, the accuracy of the classification can be improved. Moreover, the experimental results demonstrated that the number of generated images used to train the CNN was a key factor in the classification accuracy. To achieve a better classification result, we should manually select the correct generated images. This may require a substantial amount of time. In the future, we will explore methods of automatically choosing the correct generated images and establish an objective standard that more precisely evaluates the quality of the generated images.

## Figures and Tables

**Table 1:** Summary table of SAR image parameter estimation.

Distribution Type	Distribution Model	Parameter	Expression
Empirical distribution	Lognormal distribution	μ, σ	μ = $2\ln\left(m_{1}\right)-\ln\left(m_{2}\right)/2$ σ = $\sqrt{\ln\left(m_{2}\right)-2\ln\left(m_{1}\right)}$
	Weibull distribution	c, b	$\frac{\Gamma (1+2/c)}{\Gamma {\left(1+1/c\right)}^{2}}=\frac{m_{2}}{m_{1}^{2}},b=\frac{m_{1}}{\Gamma (1+1/c)}$
	Fisher distribution	M, μ	$M=\frac{m_{2}}{\left(m_{2}-m_{1}^{2}\right)-m_{1}^{2}},\mu =\frac{M-1}{M}m$
Prior distribution	Rayleigh distribution	b	$b=\sqrt{2\pi }m_{1}$
	Gamma distribution	μ	$\mu =m_{1}$
	K distribution	μ, ν	$\mu =m_{1},\nu =\frac{m_{1}^{2}}{\left(m_{2}-m_{1}^{2}\right)-m_{1}^{2}}$

**Table 2:** The settings of the CNN.

Layer Type	Image Size	Feature Maps	Kernel Size	Stride
Input layer	64 × 64	1	-	-
Convolution + ReLU	64 × 64	16	3 × 3	1
Ma× pooling	64 × 64	16	3 × 3	2
Convolution + ReLU	32 × 32	16	3 × 3	1
Ma× pooling	32 × 32	16	3 × 3	2
Fully connected	16 × 16	16	-	-
Fully connected	1	64	-	-
Output	1	7	-	-

**Table 3:** DataSet1: The original images and the images generated by PWGAN.

	The Original Images			The Generated Images		
0	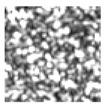	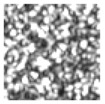	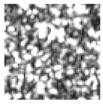	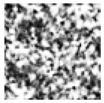	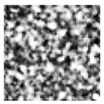	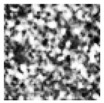
1	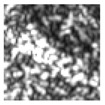	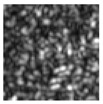	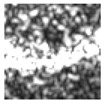	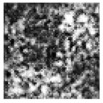	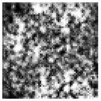	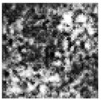
2	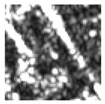	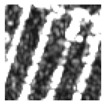	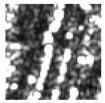	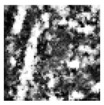	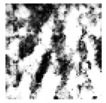	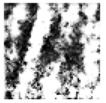
3	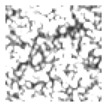	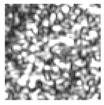	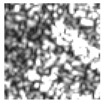	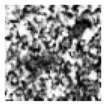	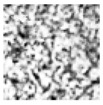	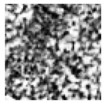
4	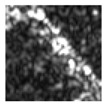	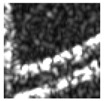	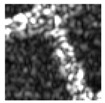	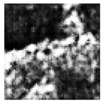	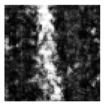	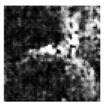
5	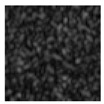	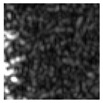	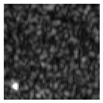	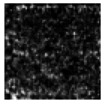	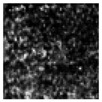	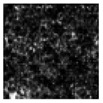
6	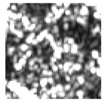	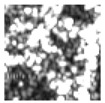	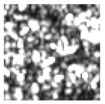	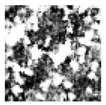	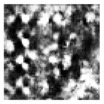	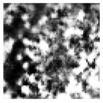

**Table 4:** DataSet2: The original images and the images generated by CNN-PGAN.

	The Original Images			The Generated Images		
0	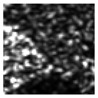	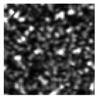	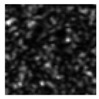	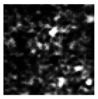	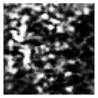	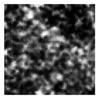
1	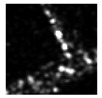	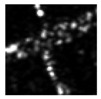	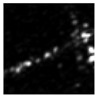	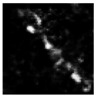	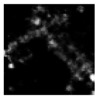	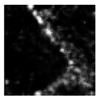
2	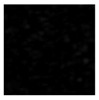	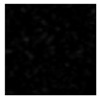	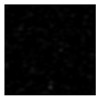	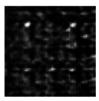	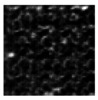	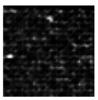
3	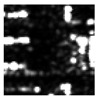	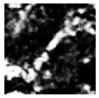	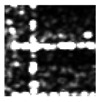	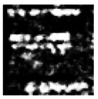	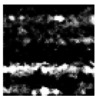	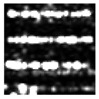
4	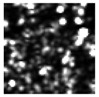	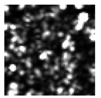	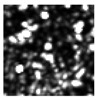	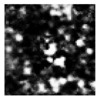	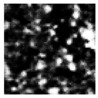	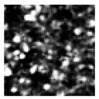

**Table 5:** The class-specific accuracy (%) and OA (Overall Accuracy) of different methods for DataSet1. DCGAN, Deep Convolutional GAN; WGAN, Wasserstein GAN.

Class	CNN	AlexNet	DCGAN + CNN	WGAN + CNN	PWGAN	CNN-PGAN
0	75	71.88	71.88	78.13	81.25	78.13
1	75	71.88	75	78.13	81.25	78.13
2	78.13	75	78.13	81.25	84.38	81.25
3	62.5	68.75	59.38	62.5	65.63	65.63
4	81.25	78.13	81.25	84.38	84.38	84.38
5	90.63	93.75	87.5	93.75	100	96.88
6	59.38	59.38	59.38	62.5	65.63	62.5
OA	74.55	74.11	73.21	77.23	80.36	78.13

**Table 6:** The class-specific accuracy (%) and OA of different methods for DataSet2.

Class	CNN	AlexNet	DCGAN + CNN	WGAN + CNN	PWGAN	CNN-PGAN
0	65.63	62.5	62.5	65.63	68.75	75
1	78.13	81.25	78.13	81.25	84.38	84.38
2	84.38	81.25	81.25	81.25	84.38	87.5
3	75	75	71.88	78.13	75	78.13
4	68.75	68.75	68.75	68.75	71.88	75.0
OA	74.38	73.75	72.5	75	76.88	80.0

**Table 7:** Classification confusion matrix (ratio) for DataSet1 by using CNN only.

	0	1	2	3	4	5	6
0	0.7500	0.0625	0.0313	0.125	0	0	0.0312
1	0.0313	0.7500	0.0937	0.0313	0.0312	0.0312	0.0313
2	0	0.0313	0.7813	0.0312	0.0937	0	0.0312
3	0.2187	0.0625	0.0313	0.6250	0	0	0.0625
4	0	0.0313	0.125	0	0.8125	0	0.0312
5	0	0.0312	0	0	0	0.9063	0.0625
6	0.0313	0.0625	0.0312	0.1250	0.1250	0	0.5938

**Table 8:** Classification confusion matrix (ratio) for DataSet1 by using the PWGAN.

	0	1	2	3	4	5	6
0	0.8125	0.0313	0.0313	0.0937	0	0	0.0312
1	0.0312	0.8125	0.0625	0.0313	0.0312	0	0.0313
2	0	0.0313	0.8438	0.0312	0.0937	0	0
3	0.156	0.0625	0.0313	0.6563	0	0	0.0312
4	0	0.0313	0.0937	0	0.8438	0	0.0312
5	0	0	0	0	0	1.0000	0
6	0.0313	0.0625	0.0312	0.0937	0.1250	0	0.6563

**Table 9:** Classification confusion matrix (ratio) for DataSet1 by using CNN-PGAN.

	0	1	2	3	4	5	6
0	0.7813	0.0313	0.0313	0.1249	0	0	0.0312
1	0.0312	0.7813	0.0625	0.0313	0.0312	0	l|0.0313
2	0	0.0313	0.8125	0.0312	0.0937	0	0
3	0.2187	0.0625	0.0313	0.6563	0	0	0.0312
4	0	0.0313	0.0937	0	0.8438	0	0.0312
5	0	0	0	0	0	0.9688	0.0312
6	0.0313	0.0625	0.0312	0.1250	0.1250	0	0.625
